# An Edge-Fog-Cloud Architecture of Streaming Analytics for Internet of Things Applications

**DOI:** 10.3390/s19163594

**Published:** 2019-08-18

**Authors:** Hung Cao, Monica Wachowicz

**Affiliations:** People in Motion Lab, University of New Brunswick, Fredericton, NB E3B 5A3, Canada

**Keywords:** IoT data streams, streaming analytics, smart parking, IoT architecture, edge computing, fog computing, cloud computing

## Abstract

Exploring Internet of Things (IoT) data streams generated by smart cities means not only transforming data into better business decisions in a timely way but also generating long-term location intelligence for developing new forms of urban governance and organization policies. This paper proposes a new architecture based on the edge-fog-cloud continuum to analyze IoT data streams for delivering data-driven insights in a smart parking scenario.

## 1. Introduction

Internet of Things (IoT) devices are usually equipped with many sensors, ranging from accelerometers and gyroscopes to proximity, light, and ambient sensors, as well as microphones and cameras. For smart cities, these devices are geographically distributed and can produce an overwhelming amount of data that poses a challenge for capturing, managing, processing and analyzing these data within a responsive acceptable time. In particular, analyzing IoT data streams generates location intelligence for many IoT applications in smart cities to engage actively with their citizens and enhance the city performance and reduce operational costs. However, this is a non-trivial process since we need a completely new IoT architecture that is capable of performing streaming analytical tasks running in parallel to provide timely approximate and accurate results.

Previous research has focused on pushing the data streams generated by IoT devices directly to a cloud environment, despite the inherited issues such as high latency, high data rates, low fault-tolerance and the unbounded order of incoming data streams [1]. Marz and his colleagues [2] proposed the Lambda Architecture, a cloud architecture that provides scalability and fault tolerance for integration of data stream processing. The main purpose of this architecture was to cope with both *“volume”* and *“velocity”* dimensions of big data, which require complex computation-intensive processes to integrate streaming analytical tasks, making it unsuitable for IoT applications [3]. Searching for simplicity, the Kappa Architecture was introduced to avoid using a batch processor by replacing it with a streaming processor able to handle data streams as an extended cache of the data flow into a cloud environment [4]. This cloud architecture may require larger in-memory storage space but it can be effective for IoT applications because it can handle fast data rates and handle retention times of the order of weeks [5].

However, IoT applications bring further fundamental and technological challenges. First, the time is ripe to rethink whether cloud computing is the only architecture able to support IoT applications, especially in the case of smart cities, where static and mobile IoT devices will be widely embedded in city infrastructure. It is worth investigating an overall orchestration of the computational resources available today that can take advantage of the edge-fog-cloud continuum to guarantee a seamless execution of automated analytical tasks without compromising the accuracy of their outcomes. Second, managing retention times between automated analytical tasks is critical for handling high/low latency of existing data life-cycles that are encountered when supporting IoT applications. However, the real advantage is not at all about latency versus throughput but rather about allowing smart cities to develop, test, debug and operate their IoT applications on top of a single analytical framework.

This paper proposes an Analytics Everywhere framework that encompasses the edge-fog-cloud continuum to support streaming analytics for maximizing the potential insights from IoT data streams. A new IoT architecture is proposed based on a conceptual framework that is particularly useful for integrating IoT devices using the edge-fog-cloud continuum. It consists of three elements that can be considered as the main criteria to take into account in order to determine whether an edge-fog-cloud environment is required by an IoT application. They can be described as follows:

- Resource capability: This element consists of organizing distributed computational nodes (i.e., cloud, fog and edge nodes) that will provide a message broker, data link, IoT device connector, data flow editor, parser, Machine Learning (ML) libraries, in-memory data storage and power for the execution of streaming tasks. Geographically adjacent compute nodes deployed at the edge, fog and cloud will be usually connected through a plethora of communication networks.
- Analytical capability: This element selects the best practice methods/algorithms for the orchestrated execution of analytical tasks that are vital to meet the requirements of IoT applications. The compute nodes are needed to perform *a priori* known analytical tasks to collect, contextualize, process and analyze data from IoT devices.
- Data life-cycle: This component describes the changes that data streams go through during the execution of analytical tasks.

The scientific contributions of this paper can be summarized as follows:

- Most of the IoT architectures rely on a cloud environment in which n-tiers of horizontal layers are designed to perform analytical tasks. Our approach proposes a new architecture based on an integrated fabric of compute nodes that are designed to work together to perform many analytical tasks, which are triggered by IoT data streams transported through an edge-fog-cloud continuum.
- Automated analytics for IoT data streams is still in its infancy and applications usually require a diverse number of outputs having different temporal granularities. There has been very little research reported on the impact of analytical tasks in the IoT architectures. The scientific contribution of our research is therefore to ascertain this impact using a smart parking scenario.

The remainder of this paper is organized as follows: Section 2 reviews the existing architectures, processing and analytical frameworks for handling IoT data streams. Section 3 introduces the main concepts of our proposed Analytics Everywhere framework. Section 4 describes the developed IoT architecture for analyzing the incoming data at anywhere and in anytime. Section 5 describes the smart parking scenario used to validate the proposed architecture. The main outcomes of the analytical tasks are shown in Section 6. Section 7 concludes our research and discusses further research.

## 2. Related Work

It is challenging to analyze vast amounts of incoming IoT data streams. Over 400 architectures have been proposed in the literature to handle incoming IoT data streams using different strategies such as *stream*, *micro-batch* and *batch processing* [5,6]. The most important issue in selecting an IoT architecture is to balance the trade off between throughput and latency. However, most approaches to handle this trade off are based on a cloud computing environment where IoT data streams are pushed to and accumulated over a long period of time and are later processed and analyzed in batches.

*Batch-oriented processing frameworks* have been efficiently used for processing large amounts of historical IoT data with high throughput but also with high latency. For example, one of the most common and widely used cloud architectures for batch-oriented processing that supports distributed storage across many clusters of commodity servers is the Hadoop MapReduce framework [7]. Another example is Spark [8] which has the ability to perform large-scale batch processing in memory using resilient distributed data sets.

Aiming to increase efficiency, *micro-batch frameworks* buffer and process IoT data streams in batch. For example, Spark Streaming restricts the batch size in a processor where each batch contains a set of events that arrived online over the batch period (regardless of the event’s time). However, it will obviously increase the time the data streams spend in the data pipeline. In contrast, *stream-oriented frameworks* typically provide time-sensitive computations but have relatively high data processing costs on a continuous stream of IoT data. Stream-oriented processing architectures usually avoid putting data at rest. Instead, they minimize the time a single tuple should spend in a processing pipeline. Some typical stream processing frameworks are Storm, Samza and Flink [9,10,11].

From an analytics perspective, IoT data streams that are accumulated for a long period of time can be analyzed in batches using traditional algorithms in machine learning and data mining such as clustering, classification, regression and dimensionality reduction, to name a few. For example, Ismail et al. [12] propose a MapReduce based mining algorithm to facilitate Parallel Productive Periodic Frequent Pattern mining of health sensors data. Ta-Shma et al. [13] also describe an attempt to ingest and analyze IoT data streams using open source components. Their simplified architecture is a combination of several instances that install an event processing framework, a batch analytics framework, a data storage framework and a message broker to handle both batch and streaming data flows. Santos et al. [14] propose an e-health monitoring architecture based on sensors that rely on cloud and fog infrastructures.

Recently, a paradigm shift has emerged in the evolution of IoT architectures aiming at analytics, software and platform configuration. Streaming analytics algorithms are being developed to extract value from IoT data streams as soon as they arrive at a computational resource. However, it is a non-trivial task to extract insights online, since the nature (or distributions) of IoT data streams change over time due to the geographical location of IoT devices [15]. Moreover, streaming analytical algorithms must work within limited resources (time and memory). Some open source frameworks for IoT data stream analytics are being developed including MOA, SAMOA and skit-multiflow [16,17,18] using only streaming processors.

Our proposed architecture is a step forward in finding a unique solution that combines the advantages of different computational resources into an integrated edge-fog-cloud fabric that is capable of capturing, managing, processing, analyzing and visualizing IoT data streams. This fabric of computational resources is designed to work towards an asynchronous approach for supporting an Analytics Everywhere framework [19] making the development, deployment and maintenance more pragmatic and scalable. By breaking down the processing and analytical capabilities into a network of streaming tasks and distributing them into an edge-fog-cloud computing environment, our proposed architecture can support streaming descriptive, diagnostic and predictive analytics.

## 3. Analytics Everywhere Framework

We propose an Analytical Everywhere framework as a conceptual model that integrates a variety of computational resources for a flexible orchestration platform that has functionality around containers. The primary goal is a seamless and automated execution of automated analytical tasks founded on a data life-cycle of an IoT application. This framework consists of three elements: Resource Capability, Analytical Capability and a Data Life-cycle which are described in more detail in the following sections.

### 3.1. Resource Capability

In general, an IoT application will require a combination of different compute nodes running at the edge, fog and/or cloud. The main criteria to take into account when selecting a compute node have been first introduced by Cao and Wachowicz [19]. They are described as follows:

- Vicinity: The geographical proximity of compute nodes to an IoT device is an important criterion to take into consideration for an IoT application. Since IoT devices can be static (i.e., deployed inside a building) or mobile (e.g., deployed in a car) and their distance to a compute node might vary, our Analytics Everywhere framework is based on the principle that IoT devices are related to everything else. Therefore, compute nodes near IoT devices are more closely related than distant ones (First Law of Geography). In particular, edge nodes should be located near the IoT devices and should use short-range networks for the data streams.
- Reachability: The time to reach a compute node via a network varies accordingly to the type of IoT devices and the communication network. Typically, if a compute node is connected to the Internet with a fixed IP address, this can be considered a highly reachable resource (i.e., it takes relatively little time to reach the compute code), rather than if it is connected using a private network and behind a NAT.
- In-memory and storage: This criterion handles the amount of data in a compute node that should be kept in memory or stored in a database. The retention time of IoT data streams is expected to vary according to the IoT application requirements as well as the available memory size. The final decision will also depend on the bandwidth and the throughput required by an IoT application. The actual amount of data which has been transmitted varies, as there could be many different factors (i.e., latency affecting throughput). The latency is clearly low at the edge due to the proximity to the IoT devices and increases as we move to the cloud.
- Computation: The amount of processing power available at a compute node for performing a set of automated analytical tasks. Taking into account the IoT application requirements can help in making a decision about which compute node to use for executing these tasks.
- Standardization: This aspect represents the most important criterion yet to be met in the implementation of IoT applications. Different standards can be applied in an IoT application ranging from network protocols and data-aggregation to security and privacy standards.

While computation and memory capabilities can increment as the analytical tasks are executing from the edge to the cloud, reachability must be always considered for an analytical task. Reachability is a critical dimension that requires analytical tasks to return results in a timely way, dependently of computational resources. Because fog nodes play the role of the intermediate resources that seamlessly integrate edge and cloud resources, the resource contention in the compute nodes and the communication links can be easily eliminated. In contrast, the proximity of the edge nodes to IoT devices can assist the necessary scaling of IoT applications, turning them into an essential computational resource for supporting near or real-time data analytics. Nevertheless, the immaturity of standards in edge resources and IoT devices are currently impeding the implementation of IoT applications.

### 3.2. Analytical Capability

We propose an Analytics Everywhere framework that can be applied to develop a variety of analytical tasks to perform descriptive, diagnostic and predictive analytics using IoT data streams. Streaming analytics are used to provide higher-level information about IoT data streams at the edge, the fog or the cloud. The aim is to generate new insights as demanded by an IoT application in order to answer the questions: *“What is happening in the real-world?”* (Streaming Descriptive Analytics); *“Why is it happening?”* (Streaming Diagnostic Analytics) and *“What will happen?”* (Streaming Predictive Analytics). The main goal of our Analytics Everywhere model is to automate *a priori* known analytical tasks that will be executed at the edge, the fog and the cloud in order to answer these questions.

**Figure 1:**
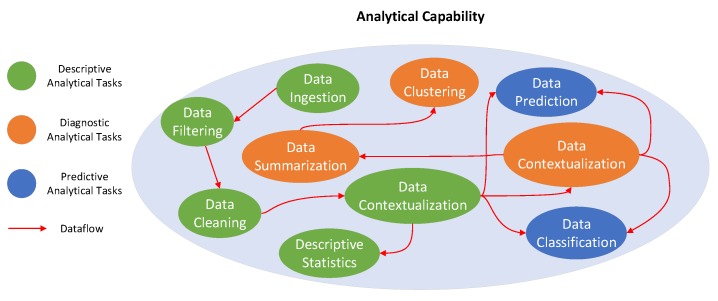
Overview of streaming tasks.

Figure 1 illustrates some analytical tasks that may be required for supporting an IoT application (green node: analytical tasks performed at the edge; orange node: analytical tasks performed at the fog; blue node: analytical tasks performed at the cloud). The analytical tasks have different levels of complexity and require a suitable data life-cycle to support multiple paths of computation ranging from data cleaning and data aggregation tasks that require a continuous stream of data, to more complex tasks such as data contextualization and data summarization tasks that require accumulated data streams for time-sensitive results. Streaming descriptive analytics may be performed at the edge, the fog and the cloud; however, we anticipate that they will more often be executed at the edge because *(i) IoT data streams have tiny volume at the edge* and *(ii) many IoT applications will prevent data from being moved to a cloud due to privacy and costs concerns*.

Streaming diagnostic analytics can be executed near to or far from an IoT device, depending on where it is more feasible to install relatively powerful computational resources. Streaming diagnostic analytical tasks are usually supported by a few on-line algorithms, stream clustering algorithms, ad-hoc queries and continuous queries. Fog and cloud resources are expected to be used to perform streaming diagnostic analytics since they provide computation, storage and accelerator resources that are more suitable than edge nodes to perform the streaming tasks. Fog and cloud computing can improve the accuracy and reduce the computational complexity of the automated tasks in near real-time. Streaming predictive analytics requires on-demand analytical tasks with high availability and rapid elasticity through the virtually unlimited resources of the cloud; the analytical tasks are expected to use a huge amount of historical IoT data that need to be processed according to the nature of IoT applications.

### 3.3. Data Life-Cycle

We expect many types of data life-cycles depending on the types of analytical tasks and compute nodes needed by an IoT application. Therefore, a data life-cycle can be either stateful or stateless depending on the orchestration requirements of an IoT application. A stateless data life-cycle treats each analytical task independently and creates output data tuples depending only on the input data tuples of that analytical task. On the contrary, stateful data life-cycles combine different analytical tasks together and create the output data tuples based on multiple input data tuples taken from those analytical tasks. Moreover, data scientists must also specify a reliability mode that can follow three approaches:

- At most once: There is no guarantee that data tuples in a stream are being handled at most once by a streaming task of an IoT application. If a failure takes place at the edge, fog or cloud nodes, no additional attempts are made to re-handle these data tuples. The assumption is that the throughput (i.e., the actual amount of data that has been transmitted between compute nodes) exceeds the maximum bandwidth. In other words, there could be different factors such as latency affecting throughput.
- At least once: The data tuples in a stream are guaranteed to be each handled at least once by all streaming tasks of an IoT application. If a failure happens, additional attempts are needed to to re-handle these data tuples. This approach may cause unnecessary duplication of data tuples in the streams but it has been widely adopted for cloud processing (e.g., Storm and Samza).
- Exactly once: Exactly once means that data tuples are guaranteed to be handled exactly the same as they would be in the failure-free scenario, even in the event of various failures.

The edge-fog-cloud continuum brings a high complexity in connecting and in orchestrating several compute nodes; therefore our Analytics Everywhere framework currently supports a stateless data life-cycle having the “at most once” approach for guaranteeing reliability and low latency for running analytical tasks of an IoT application. Two main computation paths can be found in our data life-cycle:

- Computation Path 1: analytical tasks that need user-defined Windows (batches) for accumulating data streams in order to generate outputs. Data aggregation and clustering are examples of analytical tasks that require this type of path, also called batch processing.
- Computation Path 2: analytical tasks that run using continuous data streams in order to generate outputs. Some examples of analytical tasks include data cleaning, data filtering and data duplication. This path has also been previously referred to as stream processing.

More information about how these computation paths have been applied to a Smart Parking application can be found in Section 5.2.

## 4. The Streaming IoT Architecture

**Figure 2:**
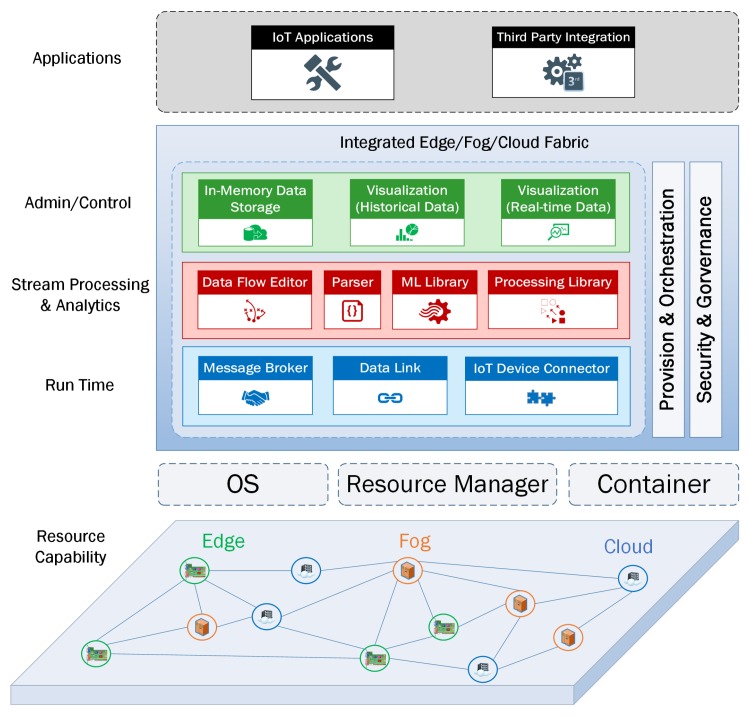
The proposed Internet of Things (IoT) architecture for our Analytics Everywhere framework.

Resource capabilities play an important role in designing an IoT architecture that relies on the edge-fog-cloud continuum for running automated analytical tasks that have a data life-cycle with streaming data tuples as input and output. We propose a geographically distributed network of compute nodes that have a combination of modules including Admin/Control, Stream Processing & Analytics, Run Time, Provision & Orchestration, and Security & Governance (Figure 2). Our IoT architecture enables micro-services to run at various compute nodes in such a way that each micro-service can perform a specific analytical task depending on which module it belongs to. It is important to point out the essential role of the Admin/Control module of our IoT architecture, since it optimizes the data flow in order to implement a data life-cycle that takes into account the individual requirements of an IoT application. Therefore, we have also integrated data management, visualization, orchestration, and security modules in our IoT architecture.

### 4.1. Stream Data Tuples

We propose an IoT architecture focusing on processing data streams which are defined as a sequence of tuples that usually contain attributes such as:

$$\left\{\left[T_{1}=\left(S_{1},x_{1},y_{1},t_{1}\right)\right],\left[T_{2}=\left(S_{2},x_{2},y_{2},t_{2}\right)\right],\ldots ,\left[T_{n}=\left(S_{n},x_{n},y_{n},t_{n}\right)\right]\right\}$$

where

$S_{n}$: is a set of attributes (i.e., measurements) obtained from an IoT device;

$x_{n},y_{n},t_{n}$: is the geographical location of an IoT device at the timestamp $t_{n}$ when a measurement has occurred.

The main characteristics of tuples can be described as one of the following:

- Each tuple in a stream arrives online. An effective architecture begins by prioritizing routing the streaming data tuples to the distributed compute nodes. This is achieved by keeping records of the ingestion times when a tuple arrives at compute nodes located at the edge, the fog or the cloud.
- An architecture has no control over the order in which a tuple arrives at a compute node. When an analytical task is automated and continuous queries are needed by an IoT application, the ingestion times play an important role in making sure all streaming data tuples implicitly belong to a user-defined window. In other words, the order of the tuples coming from an IoT device does not matter; however the order of the ingestion timestamps matter because we should not have a tuple arriving at the cloud and having an earlier ingestion timestamp from a tuple arriving at the edge.

### 4.2. Main Processing Modules

The main modules can be categorized as Run Time, Stream Processing & Analytics, and Admin/Control.

#### 4.2.1. Run Time

*Message Broker:* In our IoT architecture, the message broker is a software/middleware computer program module that reliably routes messages between clients using a formal messaging protocol and providing metadata about connected clients such as the data they are streaming and/or the actions they expose with guaranteed QoS delivery. They can also communicate with other modules, such as queries, Data Flow Editor, In-memory Databases and applications such as enterprise apps or analytical dashboards.

*Data Link:* A data link is a wrapper with a domain-specific library or functionality, that is exposed to the communication network. A data link provides an interface to access streaming data tuples from different data sources and sinks into and out of the compute nodes. It can be a device link, bridge link or an engine link. The device data links allow the capability to connect specific IoT devices together (e.g., WeMo devices, beacons, sensors). The bridge data links offer two-way communications with other publish-subscribe protocols (e.g., MQTT, AMQP, STOMP). The engine data links contain logic functions/drivers or provide access to the processes that provide specific functionality (e.g., JDBC, ODBC).

*IoT Device Connector:* This module manages the network connection between IoT devices and compute nodes. There are two main options to deploy device connector modules depending on the requirements of an IoT application: they can be described as a horizontal or as a vertical option. In the horizontal device connectors, the main components of a data stream management platform are horizontally deployed across remote nodes. In contrast, vertical device connectors not only expand their services to the edge but also scale the data stream management components to the nodes close to the IoT devices. In our architecture, we combine both horizontal and vertical options to guarantee a unique architecture based on a network of IoT devices and compute nodes.

#### 4.2.2. Stream Processing & Analytics

*Data Flow Editor:* The data flow editor is a visual data manipulation environment for wiring together IoT devices, APIs and services. It allows developers to create a data-flow model based on a set of programming blocks that perform the assigned analytical tasks when requirements are met. A data-flow model can be considered as a broker client because it can subscribe to data from different data sources and publishes results to the broker. Therefore, the data flow editor is designed to support a data-flow model to be deployed to the run time in a convenient manner.

*Parser:* The streaming data tuples can continuously bounce from one compute node to another. The goal of the parser module is to transform or serialize the tuples into a series of bytes to be stored or transmitted across a network then reverse or de-serialize them back to their original form when they reach their final destinations. Therefore, the data streams need a syntax for storing and exchanging data that is not only convenient for developers to read and write but also easy for machines to parse and generate.

*Machine Learning Library:* The main element of this module is the Online Learning Library. In contrast to batch machine learning which trains the input data, builds and evaluates the model as a bundle, the Online Learning Library is used to evaluate the current streaming data on-the-fly as they enter the compute node, and to gradually build the learning model based on the incoming data tuples over time.

*Processing Library:* This engine mainly deals with the continuous arrival of data tuples. It includes the Complex Event Processing (CEP) component and Structured Streams Processing (SSP) component to manage and transform the raw data tuples. The SSP component is used to build programs that implement operations and analytical tasks on data tuples (e.g., cleaning, filtering, updating state, defining windows, aggregating). The CEP component allows us to detect event patterns in an endless stream of events.

#### 4.2.3. Admin/Control

*Data Visualization:* This module provides two main services: the monitoring service and the exploring service. The monitoring service is used to plot real-time data whenever they arrive at our system, with the aim of early detection of abnormalities. The exploring service plots processed/historical data with the aim of assisting us with analysis and discovering new insights.

*In-Memory Data Storage:* The in-memory storage space is where the incoming data tuples and/or the results of the analytical operations reside. The storage space can be different types of in-memory databases (e.g., document-based store, key-value store) or an in-memory file system.

## 5. Validating the Proposed Architecture

In this section, we validate our proposed architecture using a smart parking application. We describe in detail the software components used to implement the main modules in the integrated edge-fog-cloud fabric. Also, the data life-cycle implementation and the IoT data streaming mechanism between each nodes in the architecture are explained in detail.

### 5.1. Smart Parking Application

**Figure 3:**
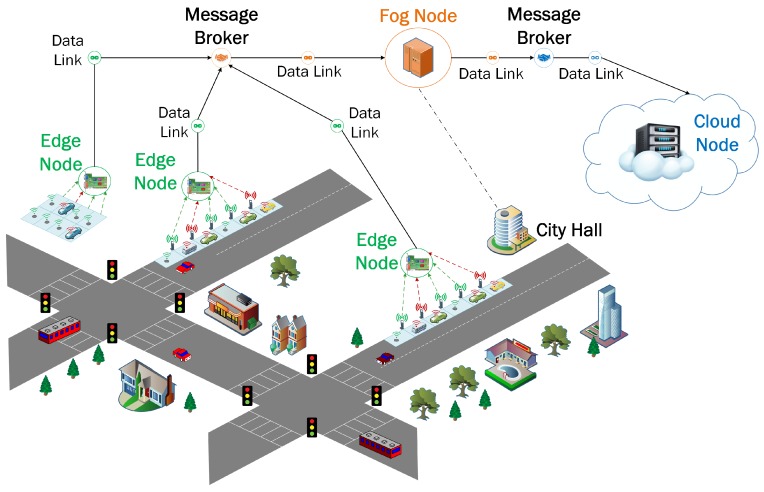
Geographical Distribution of the edge-fog-cloud nodes for the smart parking application.

A smart parking application was selected to evaluate our IoT architecture because it combines communication and information technology to help drivers find available parking spaces. Studies have shown that integrating smart parking into the city framework can shorten parking search time, reduce emissions and fuel consumption and decrease traffic congestion. The application consists of IoT data streams generated in real-time whenever a driver parks his/her car and uses the mobile application of the HotSpot Parking system which is being used in the city of Saint John, NB, Canada (Figure 3). The data streams are fetched by the edge nodes which are geographically installed close to the pay station facilities in the city. Afterward, the data streams are sent to a fog node located at City Hall. Finally, the data arrives at a Data Center provided by Compute Canada West Cloud as the IaaS resource, located in Vancouver. They are configured to communicate together as a network of nodes. The detailed specifications of each compute node are available in Table 1.

**Figure 4:**
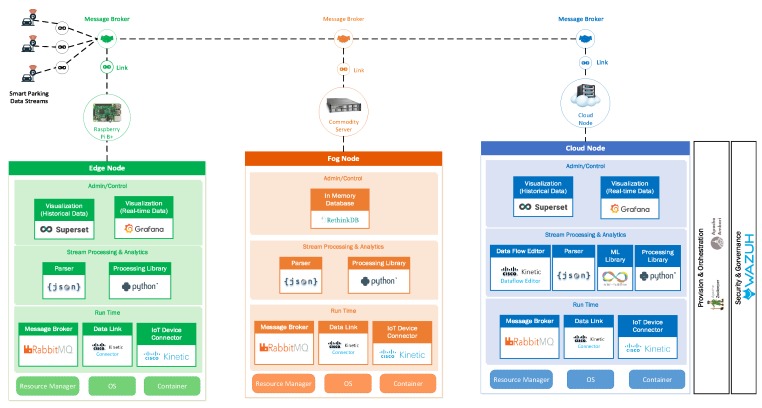
Implementation of the architecture for the smart parking application.

Different modules have been used to implement an integrated edge-fog-cloud fabric of compute nodes for the Smart Parking application. We have implemented a variety of open source modules and commercial software packages to deploy the proposed IoT architecture. Each of them plays an important role as a module in the overall architecture. The implementation is illustrated in Figure 4.

The software used for this implementation are summarized in Table 2 below.

The Analytics Everywhere framework is implemented to assist Hotspot in providing a more convenient, reliable, and professional parking service for drivers, and to assist the City of Saint John, Canada improve their parking facilities. We have selected the following analytical capabilities:

- Streaming Descriptive Analytics: What is the problem with smart parking in Saint John?
- Streaming Diagnostic Analytics: Why are these parking usage/frequency patterns an issue in Saint John?
- Streaming Predictive Analytics: What could be improved in the future?

### 5.2. Data Life-Cycle Implementation

**Figure 5:**
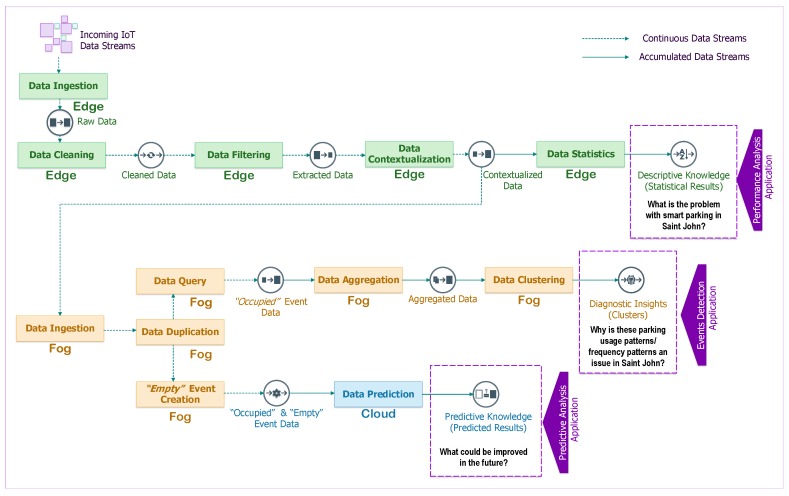
Overview of the implemented data life-cycle.

Mapping between analytical tasks and compute nodes (edge-fog-cloud continuum) for executing a data life-cycle is a non trivial task because it requires careful orchestration and a precise allocation of resources. To ease the complexity of the mapping process, a data life-cycle describes the changes that stream data tuples go through during the automated execution of analytical tasks. The Smart Parking application requires a unique data life-cycle as shown in Figure 5. Moreover, all of the analytical tasks are fully triggered and performed in an automated manner as soon as the stream data tuples arrive at any compute node.

#### 5.2.1. Analytical Tasks in Continuous Data Streams

The data ingestion task deployed at an edge node will retrieve parking data by defining a forever loop to iteratively trigger this task every 5 s. A raw streaming data tuple is considered a parking space event which will be sent to the closest edge node. The parking data streams consist of a set $\left\{T_{1},\ldots ,T_{n}\right\}$ of out-of-order tuples containing attributes in the format:

(1) $$T_{i}=\langle PE_{i},SE_{i}\rangle$$

where:

- $PE_{i}$: a specific parking event containing 4 attributes {*spot_id, length, startTime, vehicle_id*} described in Table 3.
- $SE_{i}$: a parking spot entity where the parking event is happening. It contains 3 attributes {*lat, long, spot_name*} described in Table 3.

The raw data tuples obtained after the data ingestion task will be forwarded to the data cleaning task, which consists of a sequence of operations including assessment, detection, repairing, and validation. The assessment process can detect and identify errors, redundancies, missing values, incomplete tuples, and inaccurate data fields. The tuples are re-organized, replaced, repaired or removed using adaptive integrity constraints in a dynamic sense to ensure data quality. Finally, validating the accuracy of the data tuples once they have been cleaned is an important operation before passing them to the next analytical task.

The attributes of a cleaned data tuple are later grouped into two new data fields (Parking Event and Spot Entity). Our new data tuple now becomes a set of attributes $\left\{T_{1}^{\prime },\ldots ,T_{n}^{\prime }\right\}$ in which each $T_{i}^{\prime }=\langle s_{1},\ldots ,s_{7}\rangle |i$ contains a vector of 7 corresponding attributes {*spot_id, length, startTime, vehicle_id, lat, long, spot_name*}.

We have implemented an autonomous script to logistically apply adaptive integrity constraints to handle missing attribute’s values and tuples, to remove duplicate tuples and redundant attributes, and to repair incorrect attribute values. The cleaned tuples are then transferred to the data filtering task as illustrated in Figure 5.

The data filtering automatically derives a subset of data from the original one using a set of criteria or extraction (filtering) operations. After finishing the data filtering task, the extracted data will be transferred to the data contextualization task to create new attributes and attach them to the original data tuples *T* using a contextualization operation Ψ as in Equation (2).

(2) $$\begin{array}{c} \left\{\begin{array}{c} \forall T_{i}^{\prime }\in \left(T_{1}^{\prime },T_{2}^{\prime },\ldots ,T_{n}^{\prime }\right):T_{i}^{\prime }=\langle s_{1},\ldots ,s_{7}\rangle |i\\ D=\left(T_{1}^{\prime },\ldots ,T_{n}^{\prime }\right)\overset{\Psi }{\to }\bar{D}=\left(P_{1},\ldots ,P_{n}\right)\\ \forall P_{i}\in \left(P_{1},\ldots ,P_{n}\right):P_{i}=\left(S_{i},x_{i},y_{i},t_{i},Context_{1},Context_{2},\ldots \right) \end{array}\right. \end{array}$$

The data contextualization task has been implemented at the edge to handle the current incoming data tuples and at the fog node to handle the outdated data tuples as described in Figure 5. A function was implemented to interpret the status (occupied or empty) of a parking spot whenever a driver parked his/her car.

$$f\left(T^{\prime }\right)=\left\{\begin{array}{c} T^{\prime }=T^{\prime }\cup s_{8}\;\mathrm{where}\;s_{8}=Occupied\\ T^{\prime }=T^{\prime }\cup s_{9}\;\mathrm{where}\;s_{9}=startTime+length\\ T^{\prime }=T^{\prime }\cup s_{10}\;\mathrm{where}\;s_{10}=edge\_arrivingTime \end{array}\right.$$

- Whenever a tuple arrives at the edge, we create an event label as *Occupied* and attach to the original tuple to mark that a parking spot is in use.
- We compute the *endTime* using the *startTime* and the parking duration *length*. The parking duration is the one paid by the customer.
- We also add the arriving time *edge_arrivingTime* whenever a tuple arrives at an edge node.

After the contextualization task at the edge has been executed, three new attributes $s_{8}$, $s_{9}$, $s_{10}$ are attached to the original tuple. The contextualized tuples become $T_{i}^{\prime }=\langle s_{1},\ldots ,s_{10}\rangle |i$ containing a vector of 10 attributes {*spot_id, length, startTime, vehicle_id, lat, long, spot_name,* ***event***, ***endTime***, ***edge_arrivingTime***}. This new contextualized tuple will be transmitted to the fog node where a new attribute, $s_{11}$, will be added for registering the ingestion time. At the fog, this *Occupied* data tuple is duplicated for two main purposes: (1) one copy of the *Occupied* data tuple is transmitted to accumulated data streams for further analytical tasks; (2) the other *Occupied* data tuple copy temporarily resides at the in-memory database for deducing other events.

In this smart parking application, outdated and current incoming *Occupied* data tuple are the important elements to determine the status of a parking event whenever a driver parked his/her car. We aim to infer whether an *Empty* event or an *Occupied* event is occurring at a specific parking spot.

**Figure 6:**
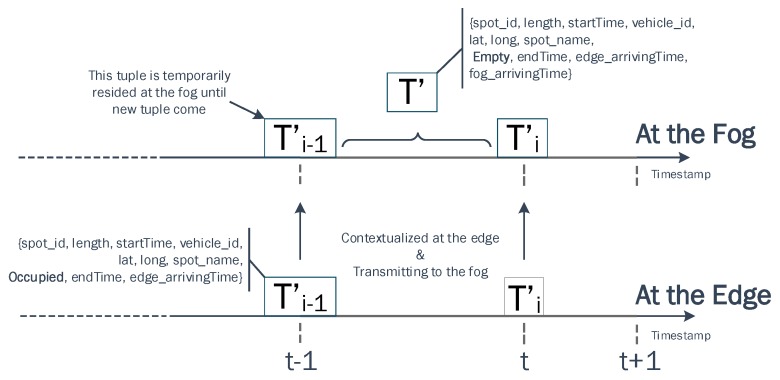
The process of computing an *Empty* event at the fog.

The *Empty* event is also computed at a fog node as shown in Figure 6. The computation consists of the following steps:

- When a contextualized tuple $T_{i-1}^{\prime }$ with an *Occupied* status arrives at the fog, it is treated as an outdated tuple and retained in database (RethinkDB) until a new tuple $T_{i}^{\prime }$ of the same parking spot arrives. To detect the changes in our real time database, we have implemented an adhoc query using ReQL language to continuously monitor the incoming tuple as follows.
**Description****ReQL Statement**Monitoring the feed if any new object changes on a tabler.**db**(‘spdb’). **table**(‘raw_historical_table’). **changes**.**run**(conn). each{|change| p(change)}
- The new tuple $T^{\prime }$ with an *Empty* status is initially computed by mirroring some static attributes from the incoming tuple $T_{i}^{\prime }$ including {*spot_id, lat, long, spot_name, edge_arrivingTime*}. Then, the *startTime* of tuple $T^{\prime }$ is assigned by the *endTime* of tuple $T_{i-1}^{\prime }$ while the *endTime* of tuple $T^{\prime }$ is assigned by the *startTime* of tuple $T_{i}^{\prime }$. The *length* of tuple $T^{\prime }$ is then computed by subtracting its *endTime* from its *startTime*. Finally, the *fog_arrivingTime* of tuple $T^{\prime }$ is attached at the end of the *Empty* tuple creation task. The following query command is used to retrieve the outdated *Occupied* tuple that temporarily resided in RethinkDB for this task.
**Description****ReQL Statement**Query the outdated *“Occupied“* tuple that temporarily resided in RethinkDBr.**db**(’spdb’). **table**(’raw_historical_table’). **without**(’id’, ’edge_arrivingTime’, ’fog_arrivingTime’). **filter**({“spot_id“: str(item[’spot_id’]), “event“: “Occupied“}). **order_by**(r.desc(’startTime’)).limit(1).run(conn)

Once the data query task and the *Empty* event creation task at the fog are completed, all outdated *Occupied* data tuples, current incoming and new tuple will contain a vector of 11 attributes $\langle s_{1},\ldots ,s_{10},s_{11}\rangle$ corresponding with {*spot_id, length, startTime, vehicle_id, lat, long, spot_name*, ***event***, ***endTime***, ***edge_arrivingTime***, ***fog_arrivingTime***}. These event data tuples will be transmitted to the data summarization task at the fog and the data prediction task in the cloud for further analytics as indicated in the lifecycle in Figure 5.

#### 5.2.2. Analytical Tasks in Accumulated Data Streams

As aforementioned in Section 3, streaming descriptive statistics task can be implemented using frequency measurement, central tendency measurement, dispersion or variation measurement, and position measurement. We chose the first approach, which implements the analytical task using frequency measurement for the smart parking application. The aim of this task is to show how often the parking event occurs by showing the parking frequency at each *spot_id* grouped by *vehicle_id*. We also analyze the parking behavior of the driver by statistically computing the parking usage of each vehicle. At the edge, the data stream can be configured to be accumulated at different time granularity (i.e., every 10 min).

The data aggregation task is executed at the fog in order to count how many times each parking spot was occupied every hour, day or month. We have implemented a Python script to trigger the data aggregation task. For example, after each hour, a set of individual summaries $\left\{Q_{1},Q_{2},\ldots ,Q_{k}\right\}$ will be produced in which each *Q* contains 4 main attributes including *{spot_id, lat, long, parking_frequency}*. The aggregated data of this task are pushed to the data clustering task for further analytics.

The aim of the data clustering task is to demonstrate how it is possible to diagnose if an incident or event occur at the fog in near real time manner. To detect an occurrence, we build an algorithm based on the Hierarchical Agglomerate Clustering (HAC) [20] approach to cluster the temporal dimensions from the incoming aggregated data. We choose to implement this unsupervised learning method at the fog because it can work independently and automatically without any human interference. The HAC method starts by partitioning a chunk of the data stream and place each data tuple into its own singleton cluster. Then, it merges the current pair of mutually closest clusters to form a new cluster. Finally, it repeats step by step until there is one final cluster left, which comprises the entire chunk of data stream.

The input of our clustering algorithm is a set of aggregated data tuples in which each data point contains 4 features *{spot_id, lat, long, parking_frequency}*. The aggregated data tuples are continuously pushing to the fog every hour. At the fog, we configure a user-defined window *weekly*. At the end of each time window, we trigger a data restructure function to sort the data so that each parking spot has not only its geo-information but also its parking frequency information at each hour during a week time window. Then, we apply the Principal Component Analysis (PCA) to select the best attributes to feed the clustering algorithm. The clustering algorithm is executed as shown in Algorithm 1.

**Algorithm 1:** Data clustering implementation for aggregated data based on Agglomerate Hierarchical Clustering approach

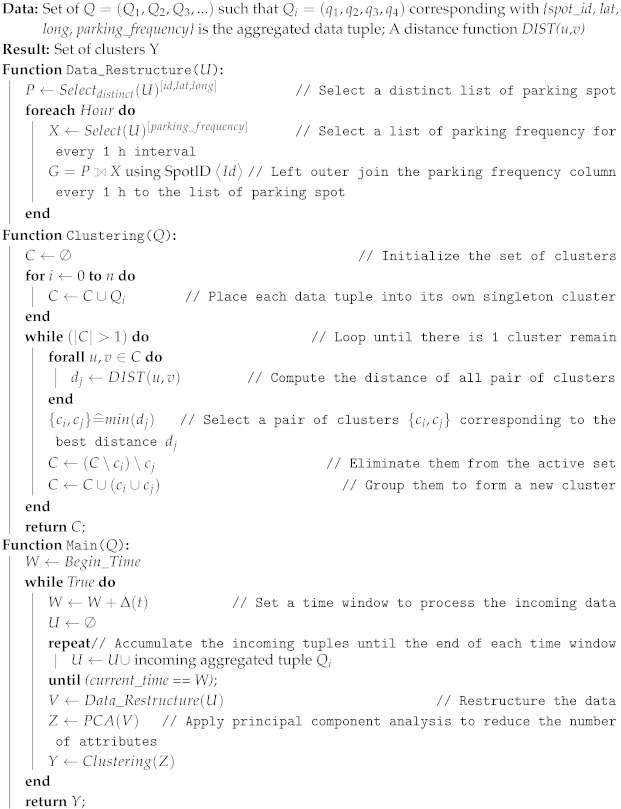



There are many criteria to measure the distance between two clusters, *u* and *v*, such as single linkage, complete linkage, average linkage, weighted linkage, centroid linkage or median linkage. In our algorithm, we use Ward linkage since it can efficiently handle noise. In this case, the distance between two clusters is measured as the following equation.

(3) $$d\left(u,v\right)=\sqrt{\frac{\left(n_{u}+n_{s}\right)d\left(u,s\right)+\left(n_{v}+n_{s}\right)d\left(v,s\right)-n_{s}d\left(u,v\right)}{n_{u}+n_{s}+n_{v}}}$$

where *u*, *v* are two joined cluster, and *s* is any other cluster; $n_{u}$, $n_{v}$ and $n_{s}$ are the size of cluster *u*, *v*, *s*, respectively.

Recently, Reference [21] proposed the algorithm Adaptive Random Forest (ARF) to make predictions on data streams. In our smart parking application, we have implemented our data prediction task for continuous incoming data tuples in the cloud based on this ARF algorithm. According to the data life-cycle in Figure 5, the contextualized data streams created by the data contextualization task will become the input data for the data prediction task. From the contextualized data stream, we receive a sequence of contextualized tuples $\left\{T_{1}^{\prime },\ldots ,T_{n}^{\prime }\right\}$ pushing from the fog in which each $T_{i}^{\prime }=\langle s_{1},\ldots ,s_{11}\rangle |i$ corresponding with {*spot_id, length, startTime, vehicle_id, lat, long, spot_name, event, endTime, edge_arrivingTime, fog_arrivingTime*}. For each tuple, we use the attribute ***event*** *= {Occupied | Empty}* as the corresponding predictive target label when it is inputted to the ARF algorithm. It is worth noting that the ARF algorithm works based on the assumption that the tuples of input data stream are independent and identically distributed (iid). In our contextualized data stream, each data tuple $T_{i}^{\prime }$ is individualistic and it does not influenced to or is influenced by tuple $T_{i+1}^{\prime }$. Also, the data contextualization task have deduce the *event* when each tuple arrive at the fog node. Therefore, the ground truth target label $T_{i}^{\prime }\langle event\rangle$ corresponding with the other attributes in tuple $T_{i}^{\prime }$ is always available before the next tuple $T_{i+1}^{\prime }$ is presented to the learning algorithm.

**Algorithm 2:** Data prediction implementation using the Adaptive Random Forest over the contextualized data stream in the cloud

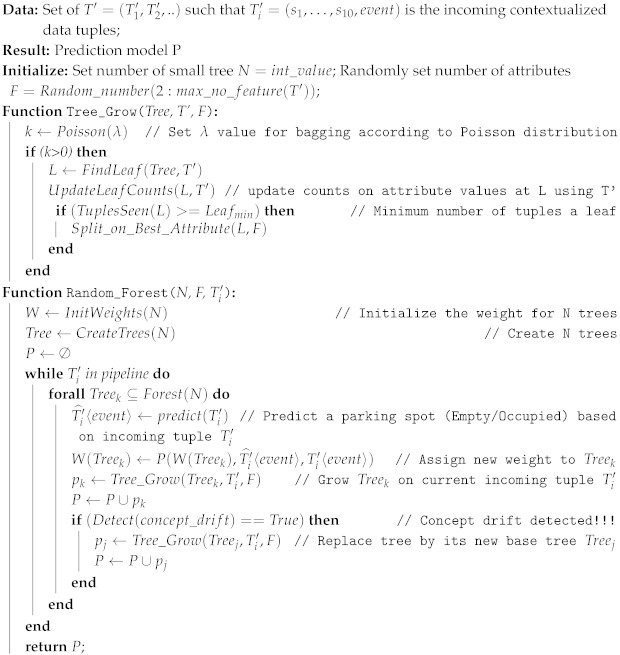



Algorithm 2 illustrate the procedure to implement the ARF algorithm in the cloud to predict the event from the incoming contextualized data stream. Different from batch random forest algorithm, where all data instances are available for training; in stream learning, training is performed incrementally as new data tuple $T_{i}^{\prime }$ is available. In the process of growing trees over the current incoming data tuple $T_{i}^{\prime }$, Algorithm 2 is able to detect whenever a concept drift happen in a tree and start to replace by its respective background tree. Performance P of the ARF model is computed according to some loss function that evaluates the difference between the set of expected target labels $T_{i}^{\prime }\langle event\rangle$ and the predicted ones $\hat{T_{i}^{\prime }}\langle event\rangle$.

### 5.3. Streaming Iot Messages

As aforementioned in Section 4, the middleware brokers are integrated into our architecture to assist us into stream the incoming data seamlessly in our system. This section aims to illustrate the details of the data streaming mechanism between the edge, the fog, and the cloud nodes in our edge-fog-cloud continuum via the AMQP protocol. This protocol allows conforming client applications at different nodes in the network of resource to communicate with each other via conforming message brokers. A node in the network of resource can play the role of a producer or a consumer. A producer is the application which can broadcast the messages to a message exchange of a broker, while a consumer is the application which can retrieve messages from the message queue. The data stream is not transmitted directly to a message queue, instead, the producer streams data to an exchange. At a broker, an exchange will route the data stream to the different queues.

**Figure 7:**
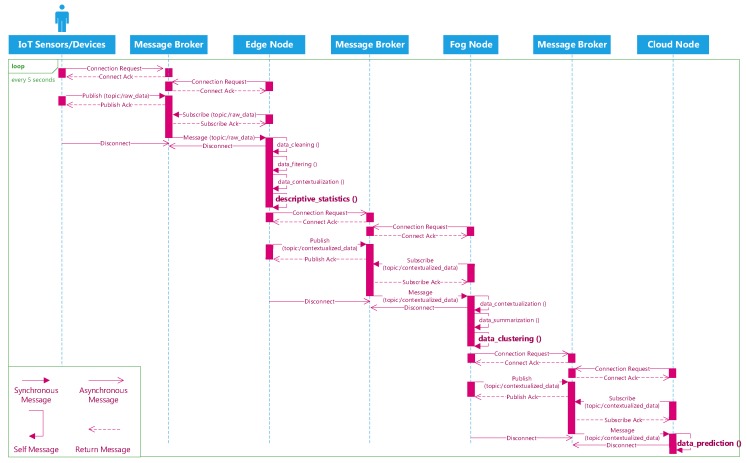
Sequence diagram for pushing the IoT data stream to the edge, fog, and cloud using Advanced Message Queuing Protocol (AMQP) protocol.

Figure 7 delineates a sequence diagram of transmitting the IoT data stream from the devices to the edge, then from the edge to the fog, and from the fog to the cloud using this protocol. First, the IoT devices/sensors connect to the first message broker and publish their generated data to the *(/raw_data)* topic. Consumer applications at the edge will connect to the same message broker and subscribe the *(/raw_data)* topic to ingest the data. At the edge, different analytical tasks can be executed before a producer application communicate with the second message broker and publish the processed data to the *(/contextualized_data)* topic. The same process happening at the fog as the consumer applications will connect to the second message broker and receive the data from the *(/contextualized_data)* topic. Again, analytical tasks are executed to diagnose the event from the data in near real time. Finally, a producer application establish a new connection with the third message broker to transmit data to the cloud. In the cloud, the consumer applications will communicate with the third message broker and retrieve data from its message queues for the predictive analytical task. Algorithm 3 depicts the sample pseudo code for the producer and consumer to exchange the data via the brokers (Watch the demo of streaming data from the edge to the fog, then to the cloud here: https://www.youtube.com/playlist?list=PL-hcE-LoSl0uMQy12yanDS8MEl5QLwp3d).

**Algorithm 3:** Sample code to stream data from Producer to Consumer

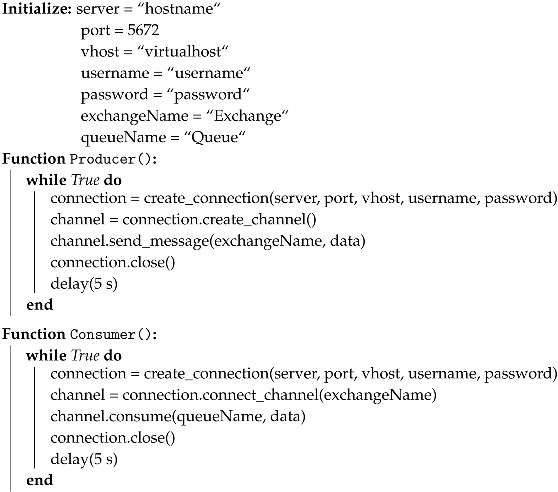



## 6. Discussion of the Results

This section describes the outcomes of the data life-cycle of our proposed Analytics Everywhere framework by showing examples of the results that emerged from our streaming descriptive, diagnostic and predictive analytics for the smart parking scenario. First, we discuss the performance of the our proposed architecture based on the latency of the data stream and the memory consumption metric. Second, we explore insights from the analytics at the edge, the fog, and the cloud.

### 6.1. Architecture Evaluation

**Figure 8:**
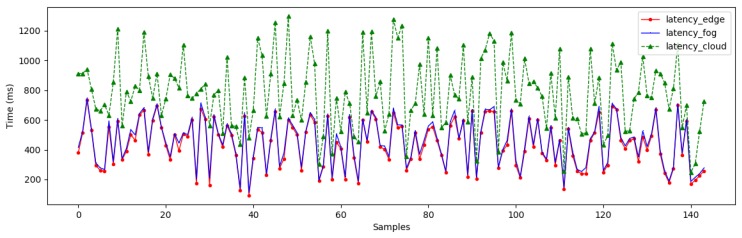
Latency Patterns.

In order to evaluate our proposed architecture, we have monitored the latency of the data streams when they arrived at our compute nodes. To compute the latency metric, we have collected samples every 10 min and registered the arrival times of the data streams at the edge, the fog, and the cloud. Figure 8 illustrates the patterns of the arrival time at different compute nodes.

As we can see, the latency at the edge and the fog are not significantly different. In contrast, there is a significant difference between them and the latency in the cloud. In fact, the latency at the edge and fog fluctuated around 150 → 800 (ms), while the latency in the cloud ranged from 200 → 1300 (ms). Although we can see similar latency patterns, a delay is clearly apparent when the data streams arrive in the cloud. This can be explained because we have deployed the edge and the fog nodes geographically close to each other using WSN in our smart parking scenario. But the data is streamed to the cloud later using the core network. These latency outcomes in Figure 8 have provided us with new insight on the crucial role of *a priori* mapping between analytical tasks with the appropriate resource capabilities.

**Figure 9:**
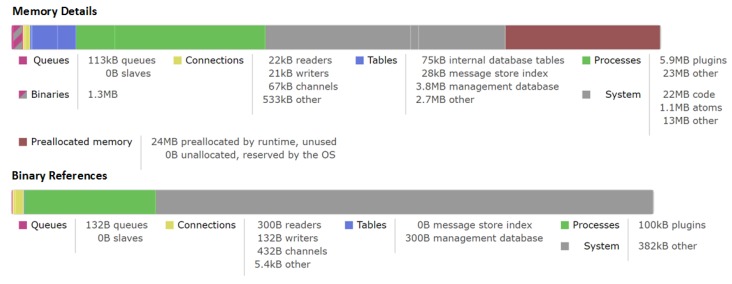
Memory Consumption Overview.

Aiming to test the ability of our proposed IoT architecture to handle the streaming traffic going through different hops in our architecture, we have computed the memory consumption details of the brokers in Figure 9. Note that the memory details shown here have been only updated on request because they could be too expensive to calculate every few seconds on a busy compute node. As we can see, the total amount of memory used was around 75 MB including allocated memory for queues, binaries, connections, tables, processes and system. It was accounted for approximate 76.5% of run time allocated for this broker during the last updated request. This result indicates that there is still a lot of room in our system to perform more heavier analytical tasks. It also shows the stability of our architecture during the IoT data streaming operations.

### 6.2. What Is the Problem with Smart Parking in Saint John?

In this section we describe the effectiveness of our architecture based on the proposed data life-cycle consisting of monitoring the usage patterns (i.e., counts of how many times the parking spot is occupied or occupancy frequency) at each parking spot every 10 min using the edge nodes to continuously process the IoT data streams.

**Figure 10:**
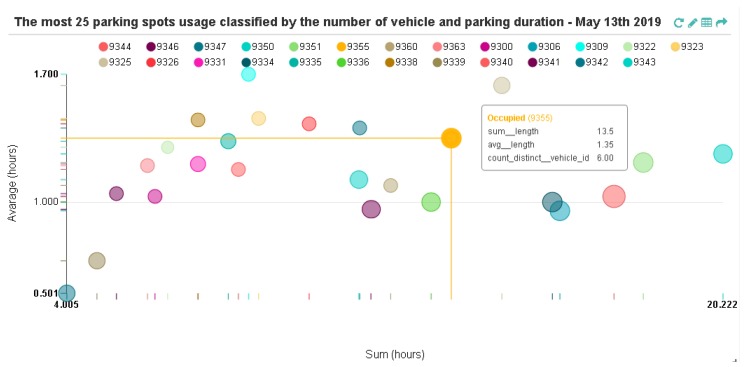
Parking usage pattern of the 25 most used parking spots.

**Figure A1:**
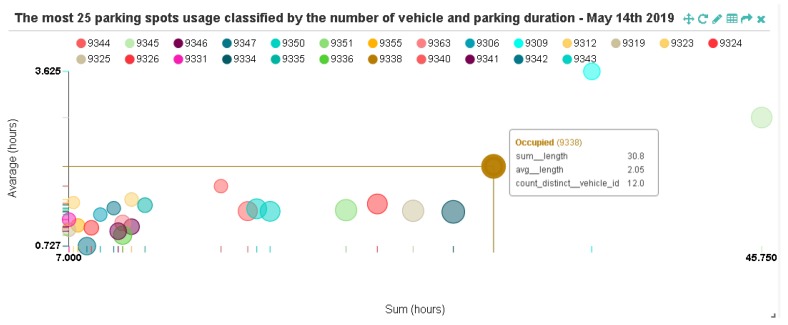
Parking usage pattern at each spot on 14 May 2019.

Figure 10 presents the usage patterns of 25 most used parking spots during a specific day of observation (13 May 2019). By comparing the total parking duration and the average parking time with the frequency of an *Occupied* event for each parking spot, we can infer that although the parking frequency is high, the average parking time at each spot is relatively low, approximately 1 h to 1.7 h. Only 2 parking spots (id 9339 & 9342) are less than 1 h. Note that if a point in Figure 10 is close to the origin coordinate, it signifies that the parking spot is usually used for short duration of time. However, the parking usage pattern was different the following day (14 May 2019); the average parking time increased to about 1.5 to 3.6 h (See Appendix A Figure A1). The data visualization analysis during a week of observation can be found in this link (https://youtu.be/YwlOWXK9F3I).

**Figure 11:**
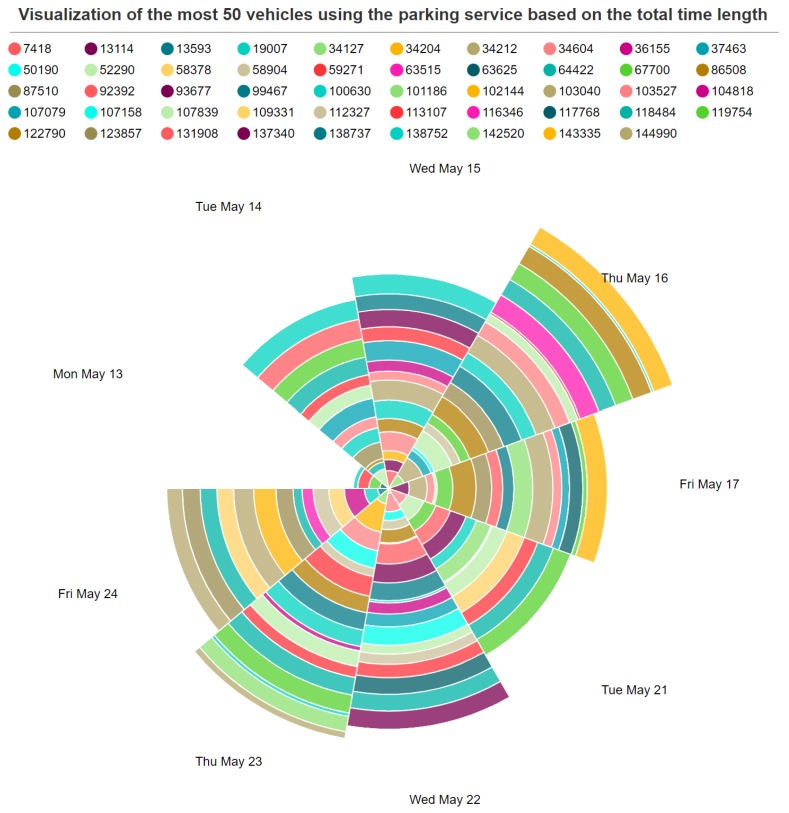
Usage patterns of the top 50 vehicles.

Figure 11 shows the statistical information about the total parking hours of the top 50 vehicles using the parking service in the city during 2 weeks of observations (13 May 2019 to 26 May 2019).

These preliminary results are already point out the under-utilization of parking spots in the city, since the frequency patterns can show often and how long the parking sports are being used.

### 6.3. Why Are These Usage Patterns an Issue in Saint John?

**Figure 12:**
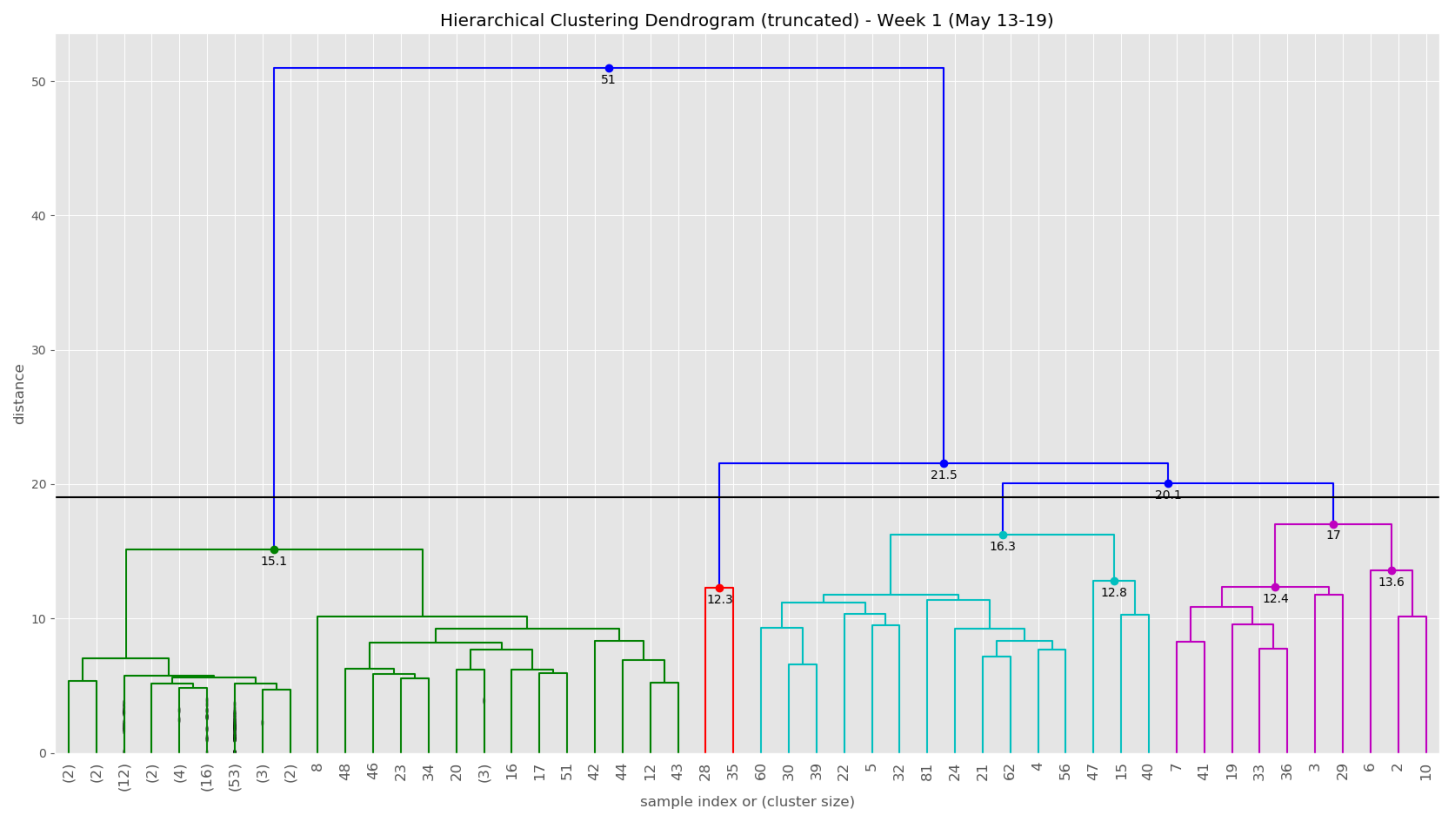
The dendrogram of the first observation week (13 May–19 May).

**Figure A2:**
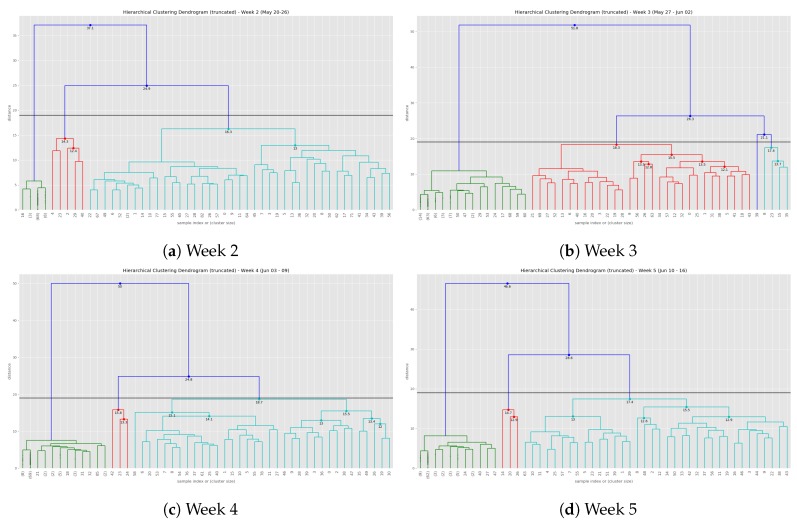
Dendrogram of clusters.

At the end of the data clustering task in Section 5.2.2, we are able to analyze to diagnose the event/incident from the clustering algorithm results. In this smart parking application, we have observed the continuous incoming aggregated data at the fog for about 5 weeks (from 13 May 2019 to 16 June 2019). Whenever an aggregated data tuple arrives at the fog, it will be accumulated and analyzed weekly. Figure 12 illustrates the dendrogram of the first observation week. The dendrogram is a tree-form visualization of the clustering algorithm showing the order and distances of merged clusters. One advancement of the HAC is that we do not need to choose a number of clusters k in advance. Instead, we can determine the number of clusters after the algorithm has been executed based on a *cut-off* distance of the dendrogram. As can be seen clearly in Figure 12, four main groups of instances are congregated into the clusters. Therefore, we have configured the cut-off distance equal to 19 (the black horizontal line in Figure 12). The dendrograms of the remaining observation weeks are depicted in Appendix B.1 (Figure A2).

**Figure 13:**
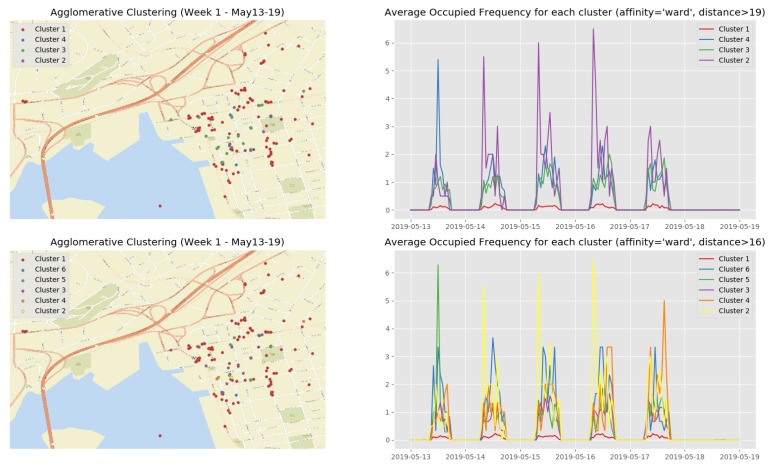
Clustering result of the first observation week (13 May–19 May).

**Figure A3:**
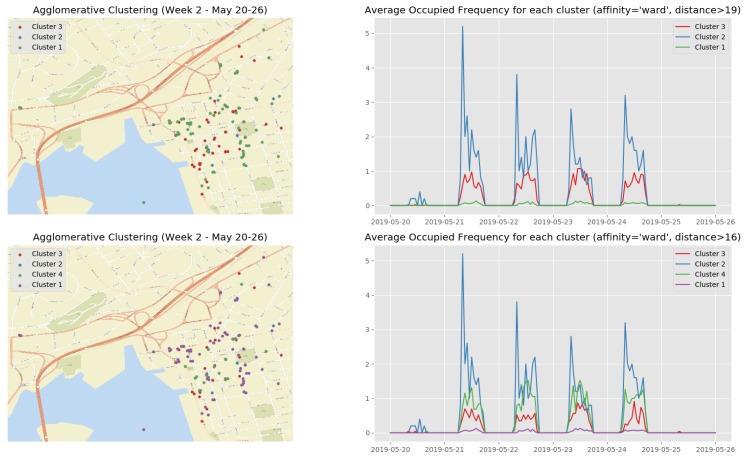
Clustering results for the 2nd week.

**Figure A4:**
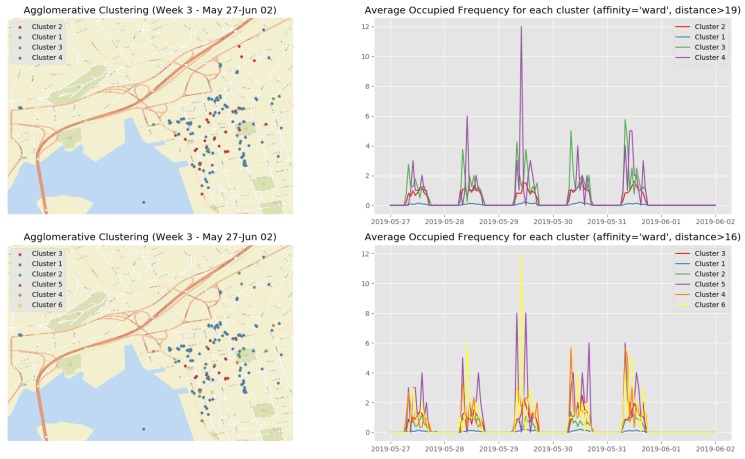
Clustering results for the 3rd week.

**Figure A5:**
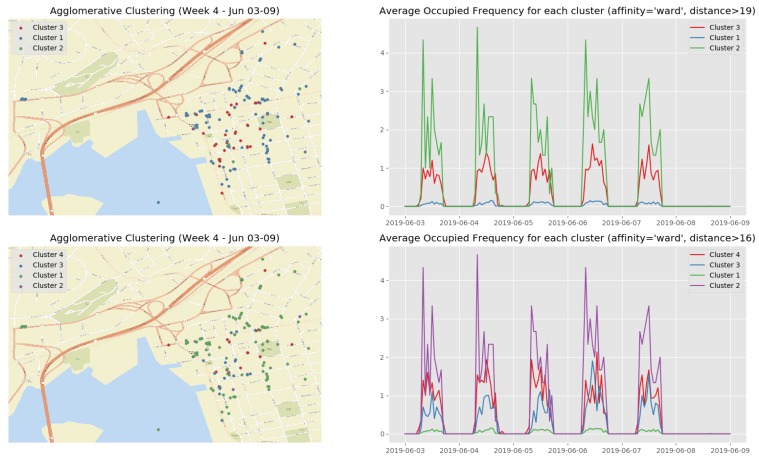
Clustering results for the 4th week.

**Figure A6:**
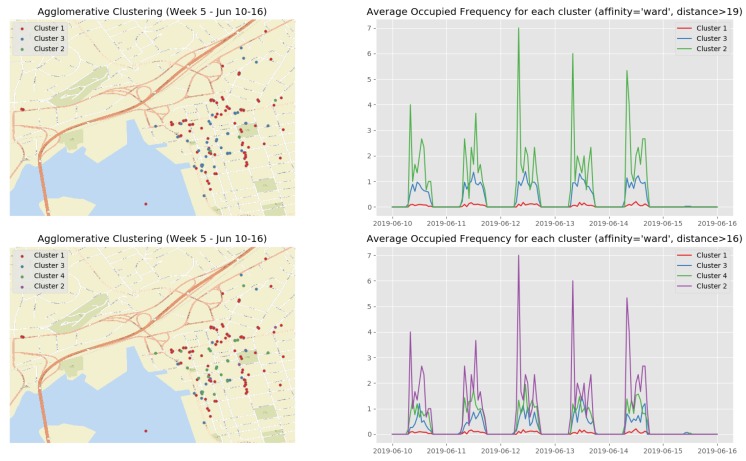
Clustering results for the 5th week.

Figure 13 show the clustering results of the temporal attributes during the first observation week. The clustering results of the remaining observation weeks are depicted in Appendix B.2 (Figure A3, Figure A4, Figure A5 and Figure A6). The top right sub figure of Figure 13 of illustrates the 4 main temporal clusters found by the HAC algorithm using a Ward distance and cut-off distance equal to 19, while the top left sub figure represents the geographical location of the parking spots for the corresponding clusters. Similarly, the bottom sub figures show the resulting temporal clusters and corresponding parking locations using the cut-off distance set equal to 16.

**Figure 14:**
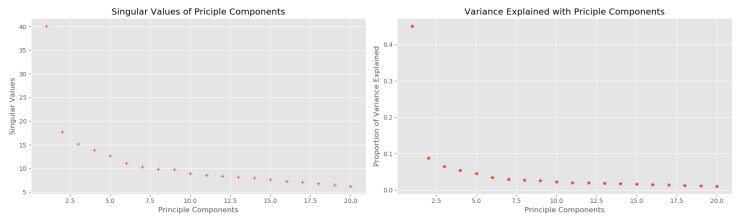
Principle Component Analysis over the aggregated data.

We used aggregated data tuples arriving at the fog hourly as the algorithm input attributes. Therefore, we have *7 days × 24 h × 1 tuple = 168 attributes* for each parking spot in the city during a week of accumulating aggregated data. Hence, we would like to perform dimension reduction using PCA on these attributes to see if any improvement on the clustering result with lower dimensionality. Figure 14 delineates the singular values of the principle components and the variance explained with these components. From this figure, we can see that the first principle component can explain approximately 60% of the data variance, and the top 5 principles together can explains nearly 80% of the data variance. Thus, we keep the first 5 components to re-implement the HAC algorithm again.

**Figure 15:**
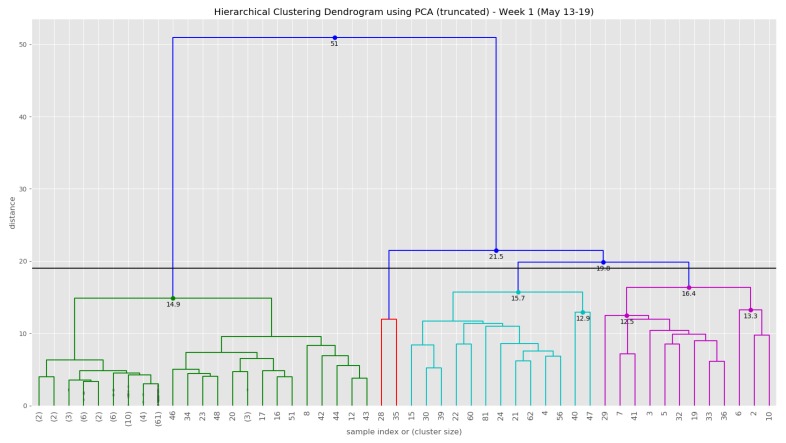
The dendrogram of the first observation week for the top 5 principle components.

Figure 15 shows the dendrogram of the first observation week when we apply the HAC algorithm on the first 5 principle components. Although there is a slight difference in the leaf nodes compared to Figure 12, the group of clusters are very similar.

**Figure 16:**
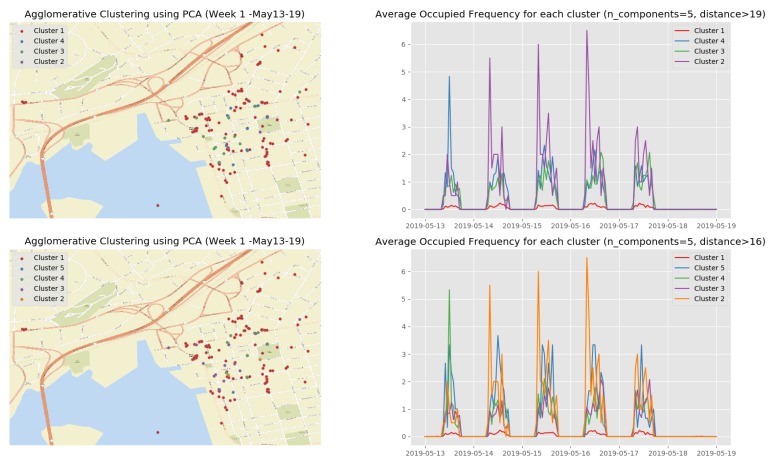
Temporal patterns of occupied/empty events that were computed at the fog node.

**Figure A7:**
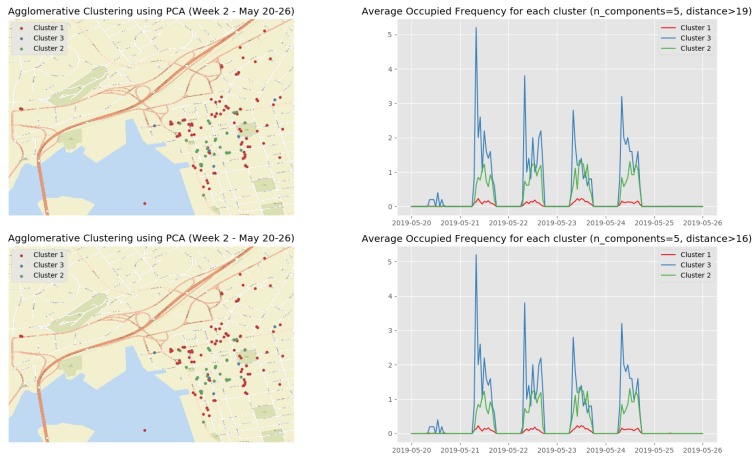
Clustering results using PCA for the 2nd week.

**Figure A8:**
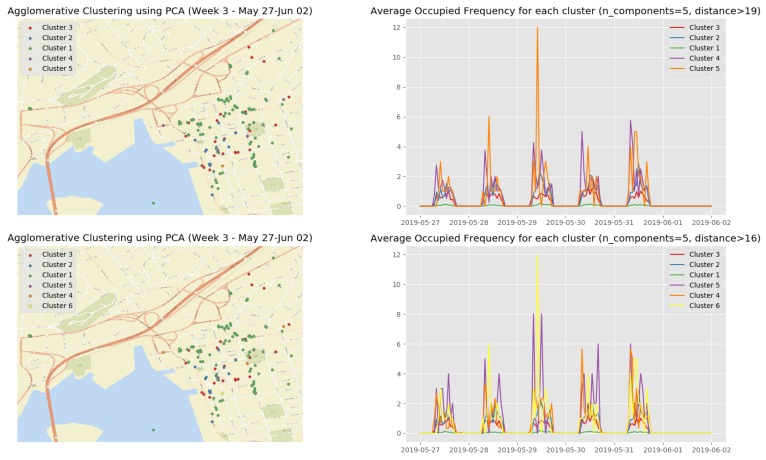
Clustering results using PCA for the 3rd week.

**Figure A9:**
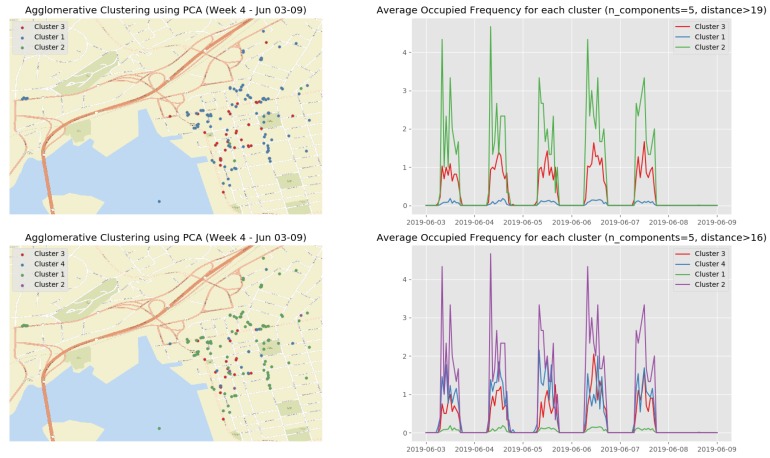
Clustering results using PCA for the 4th week.

**Figure A10:**
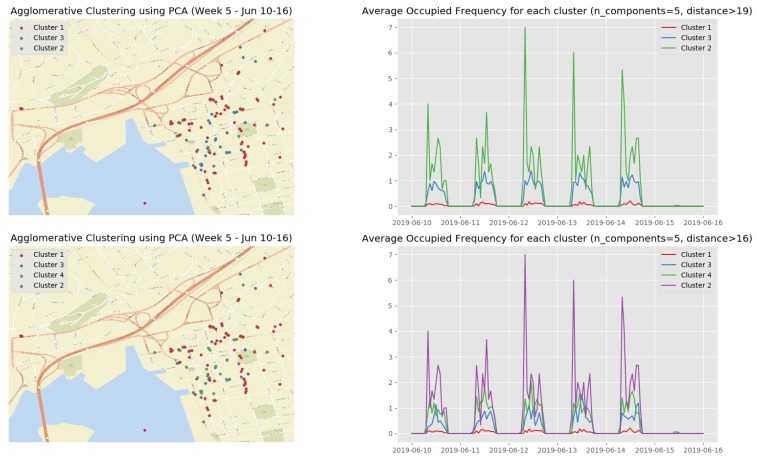
Clustering results using PCA for the 5th week.

Figure 16 shows the clustering results of the first observation week when we apply the HAC algorithm on the top 5 principle components. The clustering results on the top 5 principle components of the rest observation weeks are depicted in Appendix B.3 (Figure A7, Figure A8, Figure A9 and Figure A10) (See the evolution of the clusters here (1) https://youtu.be/XDUtc9XJWc8 (2) https://youtu.be/XDUtc9XJWc8).

*Observations & Comparisons:*

From the clustering results (see Figure 16 and Figure A7, Figure A8, Figure A9 and Figure A10), we are able to diagnose some events/incidents based on the following observations and comparisons:

- We can clearly discover 3 types of parking spots and where they are:
  - the busiest parking spots (Cluster 2 in Figure 16, Cluster 3 in Figure A7, Clusters 4 and 5 in Figure A8, Cluster 2 in Figure A9, and Cluster 2 in Figure A10) with the parking frequency from 2 to 4 times per hour, which are all in the downtown core of Saint John City.
  - the ordinary parking spots (Clusters 3 and 4 in Figure 16, Cluster 2 in Figure A7, Clusters 2 and 3 in Figure A8, Cluster 3 in Figure A9, and Cluster 3 in Figure A10) with the parking frequency from 1 to 2 times per hour, which are mainly in the downtown core of Saint John City and surrounding areas.
  - the unpopular parking spots (Cluster 1 in Figure 16, Cluster 1 in Figure A7, Cluster 1 in Figure A8, Cluster 1 in Figure A9, and Cluster 1 in Figure A10) with the parking frequency from 0 to 1 time per hour and are often located in the areas far from the the downtown core of Saint John City.
- Based on these diagnostic analytical results, we can observe that there are almost no parking event during the weekend. However, this may, in fact, be inaccurate because the smart parking facilities are free for usage during the weekend.
- Based on the clustering results, we identified that a special event/festival had taken place during second observation week. Figure A7 shows that there are not many parking behaviors on Monday, 20 May 2019. We noted that this date was a Canadian holiday, Victoria Day. However, interesting insights can be discovered from a cluster since a small number of people still paid for the parking facilities even though it was free on this day.
- Comparing the clustering results of the third observation week (Figure A8) with the other observation weeks, we discovered that an abnormal event that occurred on Wednesday, 29 May 2019 since the parking frequency reached a peek in Cluster 5. More context (e.g., a city events/festival schedule) is needed in order to explain this phenomenon.

In summary, the clustering results alone were inconclusive to identify the reasons for the under utilization of parking spots in the city. Other factors may have played a role in generating the observed clustering patterns such as parking costs and/or availability.

### 6.4. What Could Be Improved in the Future?

The incremental predictive learning model implemented in Section 5.2.2 aims to anticipate whether the status of a parking spot is *Empty* or *Occupied* in the future by training the incoming contextualized data tuples. We evaluate our model mainly based on the accuracy metric and the kappa metric. Although the accuracy metric is useful on a binary classification, it does not provide a complete picture of the performance of our predictive model. Our training contextualized data tuples contain imbalanced number of *Occupied* and *Empty* class, therefore, the kappa metric [22] is utilized alongside with the accuracy metric to avoid misleading predictive performance results. This is defined as the following equation:

(4) $$\kappa =\frac{p_{O}-p_{E}}{1-p_{E}}$$

where $p_{O}$ is the predictive model’s prequential accuracy and $p_{E}$ is the probability of an expected random chance accuracy.

**Figure 17:**
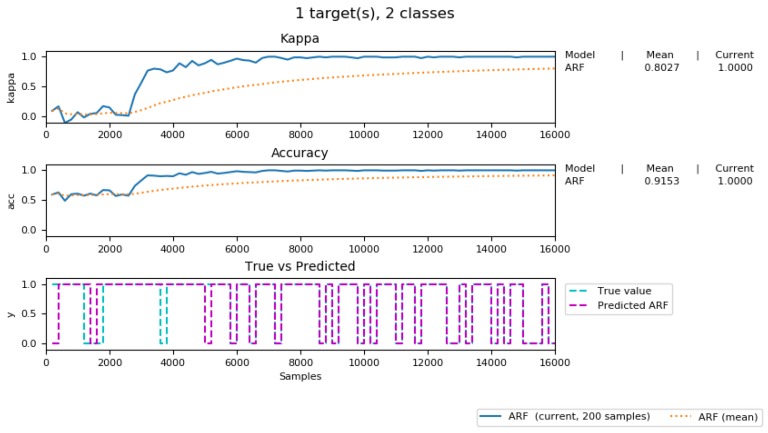
Incremental predictive learning results.

Figure 17 delineates the accuracy and kappa performance of our predictive model during the 5 weeks of incremental training (from 13 May 2019 to 16 June 2019) (See the full process of incremental training model (1) https://youtu.be/AJvxM69AFps (2) https://youtu.be/RQEaoF4WkXo). As can be seen from this figure, more accurate prediction results can be achieved by increasing the number of data tuples used produce the predictive model. Moreover, the kappa score was increased to 0.8, indicating that our predictive model was improved, compared to a random chance model.

**Figure 18:**
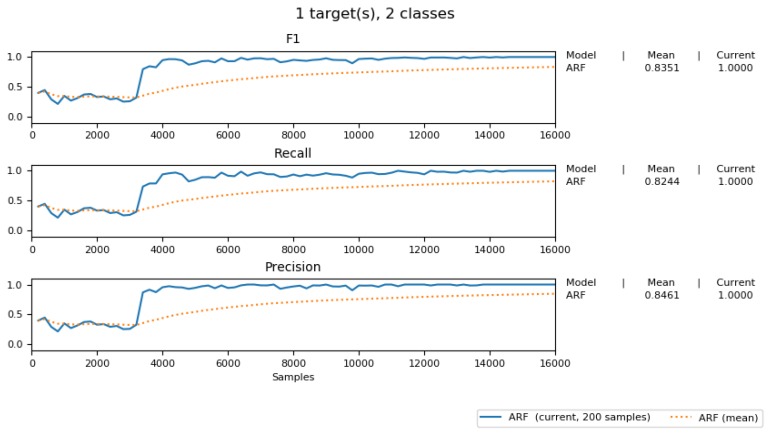
F1, Precision and Recall score of our predictive model.

Figure 18 shows the F1, Precision, and Recall score of our adaptive predicting model. It shows the high score of these three measurements and an increasing trend, similar to the one shown in Figure 17.

In general, we have built a fairly good prediction model that is able to anticipate the incoming IoT data stream. The more data to arrive in our system, the better prediction accuracy we can achieve.

## 7. Conclusions

This paper describes our preliminary results in evaluating an IoT architecture where edge, fog, and cloud resources are used to support streaming analytics for a smart parking application. The latency and memory consumption metrics have pointed out that more research is needed to develop new metrics to evaluate IoT architectures in the future. These metrics are fundamental to design the best IoT architecture in order to account for the specific requirements of the IoT applications.

Moreover, the Analytics Everywhere framework will also play an important role in generating better results for the IoT applications. The selection of analytical tasks (e.g., clustering versus classification) and performance metrics (e.g., latency) still need to be further investigated to provide more empirical results that can be used to improve our architecture.

We do not expect that one IoT architecture will fit all IoT applications. The smart parking scenario has proven that streaming analytics will always require a-priori mapping between analytical tasks and computational resources using a data life-cycle. Our future research work will also focus on developing a transfer learning process for our Analytics Everywhere framework.

Our main lesson learned after developing our IoT architecture is that if any of the edge-fog-cloud resource is considered in isolation, it would not be able to manage the data life-cycles of IoT applications without compromising the functionality or performance. However, many threats to validity of the proposed architecture using the edge-fog-computing might arise in other IoT applications. We will work with other IoT applications in smart cities, specifically those that require the analysis of time-series data and not only events.

## Figures and Tables

**Table 1:** The overview of the compute nodes.

	Edge Node	Fog Node	Cloud Node
OS	Ubuntu Mate	Window Server	CentOS 7.0 (x86_64)
CPU	ARM Cortex-A53	Intel Xeon E5-2623 v3	Intel Xeon E5-2650 v2
# of Core	4 (1.4 GHz 64-bit)	4 (3.00 GHz 64-bit)	8 (2.60 GHz 64-bit)
RAM	1 GB	30 GB	30 GB
Disk	32 GB	1 TB	1 TB
Hardware	Raspberry Pi 3 B+	Commodity Server	Virtual Machine

**Table 2:** Software used for the modules implementation.

**Run Time**	*Message Broker*	**RabbitMQ**: It is an open source streaming platform that supports different message brokers to provide fault-tolerant, scalable, high-throughput and low-latency data pipelines of queuing real time IoT data streams using a publish-subscribe mechanism.
	*IoT Device Connector*	**Cisco Kinetic**: This is a scalable, secure commercial system that can be used to extract, compute and move the data tuples to the right applications at the right time. There are three integral parts of the Cisco Kinetic platform: Edge & Fog Processing Module (EFM), Gateway Management Module (GMM) and Data Control Module (DCM).
	*Data Link*	**Cisco Kinetic Connector**: As a feature of EFM, Cisco Kinetic Connector provides a wide array of data links developed by Cisco, Third Party, and Open Source Community. It supports connectivity between compute nodes, message brokers, and IoT devices.
**Stream Processing & Analytics**	*Data Flow Editor*	**Cisco Kinetic Dataflow Editor**: This is a feature in EFM that can be used to customize, modify, and manage data flows with a graphical layout. It also offers a convenient interface to create and debug data flows.
	*Parser*	**JSON parser**: JSON objects are mainly exchanged between the computational nodes in our system. Therefore, the parser is used to encode the data structures to JSON strings and decode them back to dictionary, list, tuple, boolean or other numerical data types.
	*Stream ML Library*	**Scikit-Multiflow**: It offers main packages to assist the users with handling and learning from their data streams such as stream generators, learning methods, change detectors, and evaluation methods.
	*Processing library*	**Python**: For dealing with structured incoming data streams and detecting different data patterns, we have developed the algorithms to take action when the events happen. A variety of built-in Python libraries, such as numpy and scipy, were used to develop our algorithms.
**Admin/Control**	*In-memory Database*	**RethinkDB**: It is an open-source, distributed document-oriented database for real time changing feeds. It allows the developers to push the continuous queries to retrieve the results in real time using ReQL query language.
	*Visualization (Historical Data)*	**Superset**: Aiming to extract the insights from the historical/processed data, we have employed Superset, which is a new ongoing incubation at the Apache Software Foundation.
	*Visualization (real time Data)*	**Grafana**: It is an open-source platform capable of monitoring and analyzing the dynamic data incoming from IoT devices, which we used for our streaming real-time data.
**Provision & Orchestration**		Aiming to mitigate difficulties in managing, distributing and updating the system, we have installed Apache Ambari and Apache Zookeeper in our network of compute nodes. The Apache Ambari package is then used to configure and install the other main modules of our IoT architecture.
**Security & Governance**		For the security, we have also configured Wazuh which is an open source system for integrity monitoring, and threat and intrusion detection to protect our compute nodes. It consists of many functions such as security analytics, vulnerability detection, file integrity monitoring, and configuration assessment.

**Table 3:** The description of data tuples.

Data Fields	Attribute Name	Description
Parking Event	spot_id	The parking spot ID in the parking event table
	length	Total parking duration (hours) when a driver parks his/her vehicle
	startTime	A timestamp indicating the start time of the parking process
	vehicle_id	Vehicle ID in the parking event table
Spot Entity	lat	Latitude of the parking spot
	long	Longitude of the parking spot
	spot_name	The conventional name of the parking spot given by the City

## Abbreviations

The following abbreviations are used in this manuscript:

AMQP	Advanced Message Queuing Protocol
APIs	Application Programming Interfaces
ARF	Adaptive Random Forest
CEP	Complex Event Processing
DCM	Data Control Module
EFM	Edge & Fog Processing Module
GMM	Gateway Management Module
HAC	Hierarchical Agglomerative Clustering
IoT	The Internet of Things
JSON	JavaScript Object Notation
MQTT	Message Queuing Telemetry Transport
NAT	Network address translation
ODBC	Open Database Connectivity
OS	Operating System
PCA	Priciple Component Analysis
QoS	Quality of Service
STOMP	Streaming Text Oriented Messaging Protocol
WSN	Wireless Sensor Network
